# Using mathematical modelling to highlight challenges in understanding trap counts obtained by a baited trap

**DOI:** 10.1038/s41598-025-91581-0

**Published:** 2025-03-13

**Authors:** Omar Mazen Alqubori, Daniel Bearup, Sergei Petrovskii

**Affiliations:** 1 Department of Mathematics and Statistics, College of Science, University of Jeddah, Jeddah, 21589 Saudi Arabia; 2 School of Computing and Mathematical Sciences, University of Leicester, University Rd, Leicester, LE1 7RH UK; 3 Peoples Friendship University of Russia (RUDN University), 6 Miklukho-Maklaya St, Moscow, Russian Federation 117198

**Keywords:** Baited trap, Trap counts, Movement behaviour, Attractant strength, Individual-based model, Agroecology, Behavioural ecology, Ecological modelling, Theoretical ecology

## Abstract

Baited traps are routinely used in many ecological and agricultural applications, in particular when information about pest insects is required. However, interpretation of trap counts is challenging, as consistent methods or algorithms relating trap counts to the population abundance in the area around the trap are largely missing. Thus, interpretation of trap counts is usually relative rather than absolute, i.e., a larger average trap count is regarded as an indication of a larger population. In this paper, we challenge this assumption. We show that the key missing point is the animal movement behaviour, which is known to be modified in the presence of attractant (bait), in particular being dependent on the attractant strength. Using an individual-based simulation model of animal movement, we show that an increase in trap counts can happen simply because of changes in the animal movement behaviour even when the population size is constant or even decreasing. Our simulation results are in good qualitative agreement with some available field data. We conclude that, unless reliable biological information about the dependence of animal movement pattern on the type and strength of attractant is available, an increase in trap counts can send a grossly misleading message, resulting in wrong conclusions about the pest population dynamics and hence inadequate conservation or pest management decisions.

## Introduction

Monitoring of animal populations is required for various purposes, whenever information about the population size of a given species, population structure and health, species richness, and/or spatial population distribution is needed^1^. Common applications include disease spread^2,3^, nature conservation^4^ and pest control^5,6^. Monitoring can be performed using a variety of approaches and techniques depending on the intended purposes and the biological traits of the species^7–9^. If information is needed about the population abundance, especially for arthropod species or invertebrates more generally, monitoring is often performed using traps^10–14^. A trap is installed in a study area over a certain period of time and the animals caught are counted; based on the trap count, certain conclusions can be made about the population density in the area^11,15–19^.

Traps can have many different designs^10,12,20^. They can differ in size, shape, methods to keep the animals caught inside (e.g. by killing them or keeping alive), etc. Traps can be broadly categorised into two groups that we refer to as ‘passive’ (unbaited) and ‘active’ (baited), hence differing in the factors causing animals to get into the trap. For passive traps, trapping of an animal happens essentially by chance. For walking or crawling invertebrates, an example of such trap is a unbaited pitfall trap, which can be as simple as a hole in the ground. An animal (which we will below refer to as insect) browsing around in the vicinity of the trap may occasionally cross the rim and fall inside, hence adding to the trap count. For flying insects, a commonly used design of an unbaited trap is given by a sticky trap: a sphere or a cylinder or a stripe (or another shape) covered by a sticky substance, so that any insect that might occasionally land on the surface would remain fixed there.

Alternatively, traps can have a bait (attractant), e.g. pheromones or light, that attract insects; making their movement towards the trap more likely than in another direction. The radius at which insects respond to this bait has often been treated as the effective trap boundary of baited traps based on the concept of Effective Attraction Radius^21–23^. A baited trap can then be interpreted as large unbaited trap, and the trap counts obtained with a baited trap can be anaylsed accordingly^15,16,24^. This paradigm suggests that baited traps should produce higher trap counts than typical unbaited traps, and indeed many empirical studies^25–27^ have confirmed this intuition (though note^14^). The higher trap count can be important in some situations, in particular when the species is present but its population density is very low (so that trapping with a unbaited trap would likely result in false zeros) but early detection of the population is crucial (e.g. when monitoring a dangerous pest)^6^.

If an estimate of the population abundance is required, the information obtained from a single trap count is usually rather limited. It may prove the presence of a given species in the area, if the number of caught individuals is greater than zero, but hardly more than that (unless trapping is a part of a more complicated procedure, such as, for instance, mark-release-recapture^7^). Correspondingly, traps are often used repeatedly, so that a given trap is exposed for a sufficiently long time and is checked and emptied periodically, with caught insects being sorted and counted. This results in a sequence of trap counts. Analysis of the properties of such sequences can reveal tendencies in the monitored population, e.g. a tendency of trap counts to increase with time is usually taken as evidence that the population in the area is increasing. This has been well established for unbaited traps^17,18^. However, interpretation of trap counts obtained with baited traps is much more challenging, as any individual trap count results from a subtle interplay between the effect of population density and insect movement behaviour, where the latter can be significantly modified by the presence of the bait. Indeed, animal movement is a complex phenomenon^28^ that has several different elements and components, many of them involving animal behaviour (cf. “why move?”, “how to move?” and “when and where to move?”^28^)

Movement paths are usually described and analysed in terms of movement steps (i.e. the distance travelled between two subsequent observations) and turning angles, e.g. see^15,29–32^. An important question is what effect the presence of an attractant (e.g. pheromones or light) may have on each of the movement components. It is intuitive to expect that both the average speed of the approach (and hence the distribution of movement steps) and the frequency of turning or “zigzagging” (quantified by the distribution of turning angles) may be affected by the attractant. The exact effect, however, appears to be highly variable. Existing biological evidence^33–39^ is inconclusive, as it reveals that both components can either increase or decrease with attractant strength (and/or distance from the trap), depending on the type of bait (pheromones or light) and hence on the species. In the case of pheromone-baited traps, this uncertainty is exacerbated by the observation that the insect response depends also on the composition of pheromones, cf.^33,38^.

Considerable progress has been made during the last decade in understanding the properties of baited traps relative to unbaited traps, in particular by using a more advanced and more realistic “large unbaited trap” paradigm^15^. In several cases, this has allowed good estimates of population density to be derived from baited trap experiments^24,40,41^. However, the effects of the attractant on animal behaviour (understood here as changes in the distribution of turning angle and step size along the movement path), and how they might influence the relationship between trap counts and population density have not been fully investigated. Our study intends to partially bridge this gap in our knowledge. We achieve this through simulations using a modified individual-based model of animal movement where the distributions of movement steps and turning angles depend on the attractant strength and hence on distance from the trap. We show that such variation in behavioural response makes the population density-trap counts relationship entirely counter-intuitive and sometimes even paradoxical. In particular, trap counts may show a consistent increase over a long time interval while the total population in the area around the trap remains constant. We therefore conclude that use of baited trap without detailed biological knowledge of the effect of attractant on individual animal movement characteristics of a given species is largely meaningless and can even be, indirectly, harmful; as it can provide misleading information and hence can lead to wrong management decisions.

## Methods

### Individual-based model

Individual-based modelling approach has a long history in studies on animal movement^15,29–31,42–44^; see also^45,46^ and further references there. The idea of the approach is very simple. It uses discrete-time settings, hence assuming that the actual position of a given animal is only known at certain moments in time (which is consistent with many empirical studies, e.g.^44,47^). Knowing the animal position—say, $(x_n,y_n)$—at moment $t_n$, its ‘next’ position $(x_{n+1},y_{n+1})$ at moment $t_{n+1}$ is simulated as follows:

1 $$\begin{aligned} x_{n+1} = x_n + l_n \cos \alpha _n, \quad y_{n+1} = y_n + l_n \sin \alpha _n, \end{aligned}$$

where $l_n$ is the step size and $\alpha _n$ is the angle between the movement direction at the *n*th step and the direction of axis *x*. Here $l_n$ and $\alpha _n$ are usually regarded as random values defined by certain probability distribution functions (for a discussion of ecological implications and assumptions behind the “bugbear of randomness”, see^30^). Equation (1) can also be written in terms of the turning angle $\theta$ (i.e. the angle between the movement direction at the current and previous steps) instead of bearing using the obvious relation $\theta _n=\alpha _n-\alpha _{n-1}$.

For a given animal, steps (1) can be repeated many times resulting in a movement path $\Omega$, which is a broken line determined by the set of animal’s locations:

2 $$\begin{aligned} \Omega = \{(x_0,y_0),(x_1,y_1), \ldots , (x_n,y_n), \ldots , (x_N,y_N)\}, \end{aligned}$$

where $(x_0,y_0)$ is the initial position at $t=0$ and $(x_N,y_N)$ is the final position at some $t_N$. For a population consisting of many animals, the procedure can be repeated for each animal, hence describing how the population spatial distribution evolves with time. The movement of a population clearly results from the behaviour of the individuals in that population. We also mention here that, in a more general context, individual-based models can be regarded as a special case of agent-based models, cf.^48,49^. Using the pdfs (probability density functions) for $l_n$ and $\alpha _n$ (or $\theta _n$) with different properties^32^, one can quantify different animal movement behaviours and investigate their implications in any specific ecological context. In particular, it is straightforward to use simulations to investigate how different trap counts emerge for baseline movement patterns such as the Simple Random Walk (or Brownian Motion) or the Correlated Random Walk^45,46,50^), i.e. when space is uniform and there is no preferred movement direction.

For the sake of technical simplicity, in this study we restrict our simulations of animal movement to the 2D case. Thus, our analysis and results readily apply to movement of walking insects or small crawling invertebrates. However, generalisation of the discrete-time random walk onto the 3D case is relatively straightforward^51^. Moreover, it was shown by Ref.^51^ that, in the case of a passive (non-baited) trap, the generic pattern of trap counts is essentially the same as in the corresponding 2D case, exhibiting only relatively minor numerical differences but not any new tendencies. One can therefore anticipate that the trap counts patterns that we identify in our simulations (see below) are likely to be valid also in the 3D case and hence valid for flying insects as well.

Consider a baited trap installed at a certain position $(x_{\text {att}},y_{\text {att}})$ inside a 2D domain where animal movement is happening. The presence of of the bait creates a preferred movement direction, i.e. towards the trap. The corresponding mathematical model is known as the Biased Random Walk (BRW)^32^. An individual affected by the attractant will prefer to move towards its source, i.e. on the bearing $\kappa$ defined as follows:

3 $$\begin{aligned} \kappa = \left\{ \begin{array}{lc} \arctan \left( \frac{y_i-y_{\text {att}}}{x_i-x_{\text {att}}}\right) \pm \pi , & x_i \le x_{\text {att}}, \\ \arctan \left( \frac{y_i-y_{\text {att}}}{x_i-x_{\text {att}}}\right) , & x_i > x_{\text {att}}. \end{array} \right. \end{aligned}$$

Additionally, the attractant may act as a stimulant increasing the movement speed (or, equivalently, increasing the average step size along the movement path) and/or changing the movement persistence (the variance of the turning angle distribution). Moreover, the effect of the attractant is likely to depend on its strength, i.e. its concentration, which obviously decreases with the distance from the bait.

In order to do simulations, we have to chose specific probability density functions for the turning angle and the step size, which we assume to be truncated-normal and half-normal distributions, respectively. Thus, we arrive at the following description of the BRW:

4a $$\begin{aligned} P(\Theta ;r, d)&= \frac{N_2}{r(d)\sqrt{2\pi }}\exp \left( -\frac{(\Theta -\kappa )^2}{2 r(d)^2}\right) , \quad -\pi +\kappa \leqslant \Theta \leqslant \pi +\kappa , \end{aligned}$$

4b $$\begin{aligned} \lambda (l;s, d)&= \frac{N_1}{s(d)\sqrt{2\pi }}\exp \left( -\frac{l^2}{2 s(d)^2}\right) , \quad l\ge 0, \end{aligned}$$

where *d* is the distance between the animal and the attractant source, cf. Eq. (5) below, and $r^2$ and $s^2$ are the variances of the pdfs for the turning angle and step size, respectively. Note that $\Theta -\kappa =\alpha$, as in Eq. (1). For convenience, below we will refer to *r* and *s* as the ‘randomness parameter’ (as the direction becomes ‘less random’ with a decrease in *r*) and the ‘speed parameter’, respectively. Parameters *r* and *s* can depend on the distance from the trap *d* (for details, see section “Effects of variable attractant strength” below); in such a case, one can think of them as ‘response functions’, as they account for a variable response of movement behavior to the attractant strength. Distribution (4a) is centred on $\kappa$, hence biasing movement in that direction. Increasing the variance reduces the effect of this bias, making the choice of direction more uniformly distributed. Note that, by truncating the normal distribution (4a) at $|\Theta -\kappa |=\pi$, we neglect the contribution of large turning angles, as such turns are indeed very rare. $N_1$ and $N_2$ are normalising coefficients chosen to ensure that the total probability is one.

**Fig. 1:**
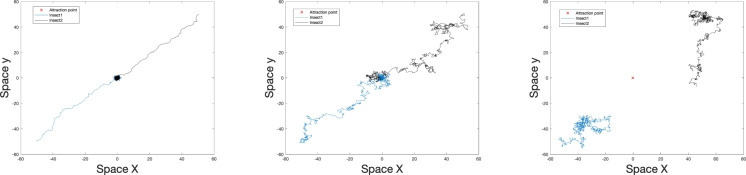
Movement paths of two animals (blue and black lines) performing a BRW over 1000 time steps with speed parameter $s = 1$ and different values of the randomness parameter: left to right, $r=0.5, 2$ and 3, respectively.

In order to demonstrate the baseline properties of the BRW, we begin with a special case where $r(d)$ and $s(d)$ are constant. Figure 1 shows how the randomness parameter affects the movement paths obtained from the BRW parameterised by Eqs. (4a) and (4b). As is intuitively expected, an increase in $r$ results in less directed movement paths, hence making the approach to the trap slower. In Fig. 1, left (obtained for $r = 0.5$), the animal moves towards the location of the attractant along a movement path that is close to a straight line. By contrast, in Fig. 1, right (obtained for $r = 3$), the path becomes very tortuous, resulting in a much slower approach.

We assume that the baited trap is a circle with radius *R*. In terms of the discrete-time simulation framework used here, this means that an animal is trapped at time $t_n$ if

5 $$\begin{aligned} d_n = \sqrt{(x_n-x_{\text {att}})^2+(y_n-y_{\text {att}})^2} < R, \end{aligned}$$

and $d_i>R$ for $i=0,1,\ldots ,n-1$. Once condition (5) holds, the movement path is terminated.

When a population of animals is moving within an area where a trap is installed, condition (5) is checked for all animals at each movement step. When an animal is caught by the trap, its movement path is terminated and the animal is excluded from the population; correspondingly, the size of remaining population (say, *N*) is reduced by 1. Denoting the number of animals caught at time step $j$ by $T_j$, one can calculate the total (cumulative) number of animals caught up to time $t_j = j \Delta t$ as follows^18^:

6 $$\begin{aligned} C_j = \sum _{k=1}^{n} T_k. \end{aligned}$$

**Fig. 2:**
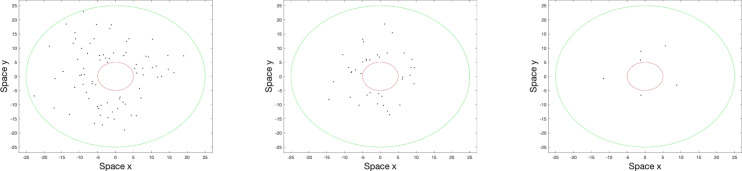
Snapshots of the spatial population distribution obtained after 30, 60 and 90 time steps (left to right) for a baited trap installed in a circular field. The edge of trap is indicated by the red circle and the edge of the field by the green circle. The simulation parameters are as follows: field radius $L = 25$, trap radius $R=5$, initial population $N_0 =78$, speed parameter $s = 1$ and randomness parameter $r=0.5$.

Correspondingly, the size of the remaining population is given by $N_j = N_0 - C_j$ where $N_0$ is the inital population size. Thus, in case the area is closed (i.e. there is no migration from outside), as a result of trapping the remaining population declines monotonously with time. This is readily seen in simulations. An example is shown in Fig. 2 where movement of a population of initial size of $N_0=78$ individuals is simulated in a circular field with a circular trap located at its centre, i.e. at $(x_{\text {att}},y_{\text {att}}) = (0,0)$. At time $t=0$, the population is uniformly distributed over the field. Clearly, the number of animals remaining in the field gradually decreases with time. Note that, over time, the spatial distribution of animals becomes nonuniform; with individuals becoming more concentrated in the vicinity of the trap rather than further away. This is clearly the effect of the attractant (bait) as animals are more likely to move towards the trap than in another direction. Interestingly, in the case of an unbaited trap, where animals move without a preferred direction, the effect of trapping is exactly opposite; resulting in a lower population density in the vicinity of the trap and a higher density away from it^17,18^.

**Fig. 3:**
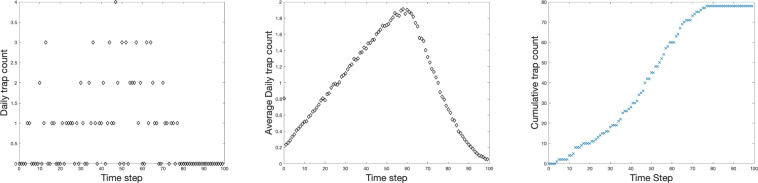
Baited trap counts obtained at each moment of time. Parameters are the same as in Fig. 2. Left: individual trap counts after each time step, $T_j$. Middle: Average individual trap counts across $3000$ realizations of the system. Right: Cumulative trap counts after each time step, $C_j$.

The corresponding trap counts obtained for the above system are plotted in Fig. 3 (where for convenience of interpretation we refer to the unit time step as a ‘day’). We note first that the number of individuals caught at each time step $T_j$ is essentially a random variable (cf. Fig. 3, left), which is, of course, not surprising given the random walk of individual animals. The distribution of probabilities for each trap count (which in this particular case varies between 0 and 4) changes over time, so that higher counts become more likely at some intermediate time. Arguably, it reflects the change in the spatial distribution of animals in the vicinity of the trap. This change in the ‘daily’ trap counts over time is readily seen if the results are averaged over a number of realisations of these inherently stochastic dynamics, see Fig. 3, middle. Interestingly, the average ‘daily’ trap count increases steadily over a significant period of time, in spite of the fact that the size of the population in the area where the trap is installed is actually gradually decreasing. Note that this pattern in the trap count dynamics is quite different from that obtained for an unbaited trap where the average trap count always (for the population initially distributed uniformly) show a monotonous decrease with time^17^.

Another way to see a clearer pattern in the trap counts over time is to consider the cumulative trap counts $C_j$ instead of individual trap counts; see Fig. 3 (right) obtained for the simulation data shown in Fig. 3, left. The effect of stochasticity is much less apparent in this case, so that the cumulative trap count dependence on time is close to a smooth curve. Note that the cumulative trap counts initially grow slowly but the rate of growth increases with time, resulting in a steeper slope of the curve, where the maximum growth rate corresponds to the maximum average daily trap count (shown in Fig. 3, middle).

In conclusion to this section, we mention that in our model we consider animal movement in a finite domain with an impenetrable boundary (hence excluding the possibility of insect immigration). Clearly, this means that the population size cannot increase; as a matter of fact, it gradually decreases with time as a result of trapping, so that in a large time limit all individuals are caught. While this situation may directly correspond to animal trapping under laboratory conditions^52^, in terms of real-world open systems this outcome is unlikely to happen. Correspondingly, it means that the exact predictions of our model are limited to a certain intermediate timescale. On a longer timescale, our model gives a lower bound for trap count values that would be obtained in a system where the population size is much larger than the cumulative trap count over the time of trap exposure, so that the decrease in population size due to trapping can be neglected. 

### Effects of variable attractant strength

In the simulations presented in the previous section, the strength of the attractant was assumed to be constant all over the domain where the baited trap is installed. However, this is not entirely realistic. Regardless of the nature of the attractant, e.g. pheromone or light, its strength (respectively, pheromone concentration or light intensity) will decay with the distance from its source, i.e. with the distance from the trap. Therefore, any change in the movement behaviour caused by the presence of attractant is likely to depend on the distance too. Correspondingly, in this section, we will consider a more realistic model (4) where *r* and *s* are functions of the distance from the trap. Our purpose is to reveal whether the important preliminary finding of the previous section, i.e. that trap counts can increase steadily over time even if the monitored population is actually decreasing, changes for a more realistic choice of *r* and *s*.

### Parameterisation of distance dependence

We take distance from the trap as a proxy for attractant strength, and construct archetypal models to describe how an individual’s behaviour may vary within the domain. Unfortunately, there is only rather meager biological information about the type of dependence of movement components on the attractant strength and hence on the distance^34,37,39^. We therefore have to consider a number of different hypothetical cases based on some generic biological argument.

#### Parameterisation of the turning angle

We assume that, in the absence of attractant, there is no any preferable direction. Thus, at a sufficiently large distance from the trap, the distribution of turning angle should be approximately uniform over the circle, i.e. *r* should be sufficiently large. However, as the distance decreases, it is intuitive to expect that movement should become more directional towards the source of attractant (known as positive phototaxis in case of light-baiting, cf.^53^) and there is indeed compelling biological evidence of that^34,37,54^; hence *r*(*d*) should be an increasing function. To account for this possibility, we consider the following parameterisation:

7 $$\begin{aligned} r_1(d) = a_1 \exp (b_1 d), \end{aligned}$$

where $a_1$ and $b_1$ are positive parameters.

Note that the graph of $r_1(d)$ is a convex curve, which means that the effect of attractant decays with distance at accelerating rate. In order to check whether such effects of the shape are important, we consider an alternative parameterisation that is given by a concave curve, namely, the following function:

8 $$\begin{aligned} r_2(d) =\frac{a_2 d}{1 + b_2 d}, \end{aligned}$$

where $a_2$ and $b_2$ are positive parameters. In this case, the effect of attractant decays with distance at a decelerating rate.

We also consider a more subtle case of the movement dependence on the attractant strength (hence turning angle’s dependence on distance). Several studies reported that, once the attractant strength becomes too high, insects may become disoriented and hence their movement may become less directional, more erratic^54,55^. In case of light, this effect is sometimes referred to as insect dazzling^53^. Correspondingly, we consider the case where movement becomes more directional for an increase in the attractant strength from low to some intermediate value (as in Eqs. (7) and (8)) but becomes less directional for a further increase in the attractant strength, so that the variance of the turning angle distribution reaches its minimum at some intermediate distance:

9 $$\begin{aligned} r_3(d)&= J_3 (d-a_3)(d-b_3)+1, \end{aligned}$$

where $a_3$, $b_3$ and $J_3$ are positive parameters. Obviously, the minimum is reached at $d=0.5(a_3+b_3)$ and can be easily calculated as $\min {r_3}=1-\frac{1}{4} J_3(a_3-b_3)^2$. Note that, since due to its meaning $r_3(d)\ge 0$ for any *d*, $\min {r_3}\ge 0$ and hence the above expression brings corresponding restrictions on parameter values, i.e. $|a_3-b_3|\sqrt{J_3}\le 2$. This is taken into account when choosing parameter values for simulations, see Table 1.

In order to look into possible effects of the details of a particular parameterisation of turning angle on trap counts, we also consider a more complicated dependence on the distance:

10 $$\begin{aligned} r_4(d)&= \frac{J_4 \exp (-a_4 d)+q \exp (b_4 d)}{1+\exp (b_4 d)}, \end{aligned}$$

where $a_4$, $b_4$, *q* and $J_4$ are positive parameters. For appropriately chosen parameters, similarly to $r_3(d)$ in (9), $r_4(d)$ can have a minimum at some intermediate distance. However, with an increase in *d*, $r_4(d)$ eventually approaches its asymptotic value *q*, instead of the unbounded growth as in (9).

**Fig. 4:**
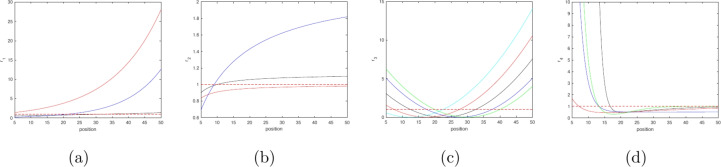
Dependence of the randomness parameter *r* on the position in the field (distance from the trap) for different parameter values. From left to right, $r_1$ to $r_4$ (Eqs. (7) to (10), respectively); parameter values are given in the first eight rows of Table 1. The colours—black, red, blue, cyan and green—correspond to the rows in each of the boxes in Table 1.

**Fig. 5:**
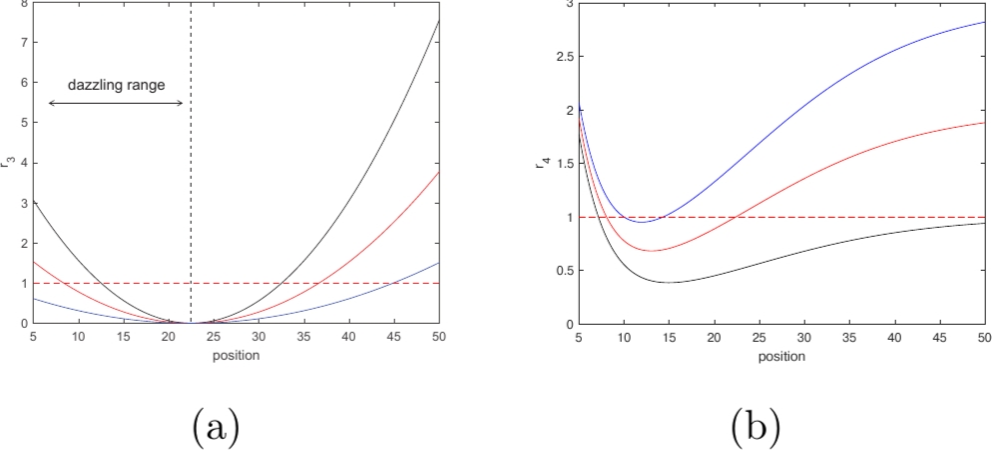
Dependence of the randomness parameter *r* on the position in the field (distance from the trap) in case insect movement becomes disoriented with an increase in the attractant strength (“dazzling effect”) for different parameter values: (**a**) for $r_3$ (cf. Eq. (9)), (**b**) for $r_4$ (cf. Eq. (10)). Parameter values are given in the last three rows of Table 1.

In order to capture the full effects of the different behavioural types described above, we need to ensure that the parameter values chosen produce sufficiently distinct response functions. In particular, a response function should include regions where both directional and random behaviour takes place. We note that the transition from strongly directional behaviour to more random behaviour occurs when the randomness parameter is close to one, see Fig. 1. As such, we can obtain a suitable range of behaviours if the randomness parameter function crosses this threshold between the trap and outer boundaries of the field. Examples of suitable parameters for Eqs. (7)–(10) can be found in Table 1 and plots of these functions in Figs. 4 and 5.

**Table 1:** Parameter values resulting in a representative variation in the movement behaviour (as is quantified by variation in the randomness parameter) over the space, i.e. the distance from the trap).

Response function	$a_i$	$b_i$	$q$	$J_i$	Response function	$a_i$	$b_i$	$q$	$J_i$
$r_1$, Eq. (7)	0.5	$\tfrac{a_1}{R^2}$	$-$	$-$	$r_2$, Eq. (8)	0.9	0.8	$-$	$-$
Same	1	$\tfrac{a_1}{3R}$	$-$	$-$	Same	1	1	$-$	$-$
Same	0.14	0.09	$-$	$-$	Same	0.2	0.09	$-$	$-$
$r_3$, Eq. (9)	15	30	$-$	0.01	$r_4$, Eq. (10)	0.8	0.1	1	0.01
Same	10	25	$-$	0.01	Same	0.3	$\tfrac{a_4}{R}$	1	0.01
Same	5	50	$-$	0.01	Same	0.5	0.001	1	0.01
Same	20	40	$-$	0.01	Same	0.5	$a_4^2$	1	0.01
Same	5	20	$-$	0.01	$-$	$-$	$-$	$-$	$-$
$r_3$, Eq. (9)	15	30	$-$	0.01	$r_4$, Eq. (10)	0.3	0.1	1	0.01
Same	15	30	$-$	0.005	Same	0.3	0.1	2	0.01
Same	15	30	$-$	0.002	Same	0.3	0.1	3	0.01

The lower section of the table is separated from tjhe middle part in order to emphasize on variation of parameters $J_3$ and *q*. The corresponding functions $r_k(d)$ ($k=1,2,3,4$) are shown in Figs. 4 and 5.

#### Parameterisation of the step size

The presence of an attractant is known to affect the speed of animal movement, e.g. see^34,37,39^, although the existing evidence is rather meagre and only available for a few species of flying insects but not for walking insects or other invertebrates. Moreover, the exact effect of the attractant varies significantly depending on the nature of the bait. For pheromones, an increase in their concentration was observed to result in a decrease in the speed of the flight^34,39^. However, in case of light baited traps, an increase in light intensity was found to have an opposite effect resulting in an increase in the speed of the flight^37^.

In our parameterisation of animal movement, an increase in the movement speed means that movement steps of a larger size become more likely. Given our assumption that the distribution of step size is described by a half-normal distribution, see $\lambda (l;s, d)$ in (4), we take that into account by considering an increase in the distribution variance. For the given pdf as in (4), an increase in *s* automatically leads to an increase in the mean step size $<l>$ and hence to an increase in the average movement speed.

Given the uncertainty in the available biological evidence, we consider several hypothetical dependencies of *s* on the distance from trap (and hence on the attractant strength):

11 $$\begin{aligned} s_1(d)&= \frac{c_1}{\sqrt{d}}+h_1, \end{aligned}$$

12 $$\begin{aligned} s_2(d)&= \frac{c_2}{d}+h_2, \end{aligned}$$

13 $$\begin{aligned} s_3(d)&= \frac{c_3}{d^2}+h_3, \end{aligned}$$

where $c_{1,2,3}$ and $h_{1,2,3}$ are positive parameters. Note that all three functions (11)–(13) implicitly assume that the movement speed tends to decrease with the distance (so that the variance reaches its minimum value [respectively, $h_1$ or $h_2$ or $h_3$] when distance becomes very large), in agreement with the known effect of light on some flying insect species^37^. However, the rate of decrease is different, being lowest for (11) and fastest for (13).

Note that, since light is a physical quantity, the decay of its intensity with distance is controlled by physical laws. In particular, in an ideally transparent air (neglecting light absorption), for a light source of a simple design, the decay rate of the light intensity should be proportionate to $d^{-2}$, cf. Eq. (13). In less transparent air, where the absorption cannot be neglected, the decay rate should be somewhat faster. By considering a slower decay rate, i.e. (11) or (12), we therefore assume that the movement speed is not simply proportionate to the light intensity but is moderated by animal’s behavioural response, which seems to be in general agreement with available observations^37^.

Similarly to the previous section, in order to account for the possibility of a more complicated behavioural response to the attractant strength, we extend the list of functions (11)–(13) by adding another case where the variance of the step size distribution reaches its maximum at a certain intermediate strength and hence at some intermediate distance $d_{max}$ from the trap:

14 $$\begin{aligned} s_4(d) = c_4 \exp \left( -\frac{(d-d_{max})^2}{h_4^2}\right) , \end{aligned}$$

(where $c_4$, $h_4$ and $d_{max}$ are positive parameters) so that maximum movement speed is achieved at $d=d_{max}$.

**Fig. 6:**
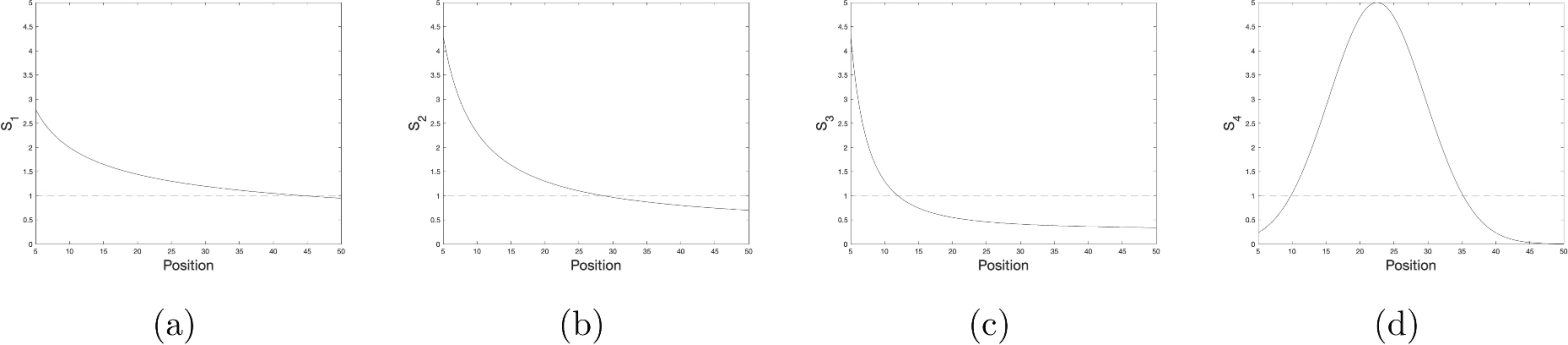
Dependence of the step length parameter *s* on the position in the field (distance from the trap) for different parameter values. From (**a–d**), $s_1$ to $s_4$ (Eqs. (11) to (14), respectively); parameter values are given in Table 2..

In order to make cases (11)–(14) comparable, we choose the parameters $c_{1-4}$ and $h_{1-4}$ in such a way that the maximum value is approximately the same for all four functions $s_1(d)$, $s_2(d)$, $s_3(d)$ and $s_4(d)$. We also require that, in all four cases, the minimum value falls to a sufficiently low value (below one) within the considered range $R\le d\le L$, hence making the range of expected behavioural responses sufficiently broad. The parameter values that we will use in simulations are given in Table 2 and the plots of the corresponding functions are shown in Fig. 6.

**Table 2:** Parameter values resulting in a representative variation in the movement behaviour (as is quantified by variation in the step length parameter) over the space, i.e. the distance from the trap).

Response function	$c_i$	$h_i$	$d_{max}$	Response function	$c_i$	$h_i$	$d_{max}$
$s_1$, Eq. (11)	6	0.01	$-$	$s_2$, Eq. (12)	20	0.3	$-$
$s_3$, Eq. (13)	100	0.3	$-$	$s_4$, Eq. (14)	10	5	22.5

The corresponding functions $s_m(d)$ ($m=1,2,3,4$) are shown in Fig. 6.

## Results

In this section, we use the individual-based model described above (see section “Individual-based model”) with the movement characteristics depending on the distance from the trap (to reflect their likely dependence on the attractant strength), as is described in the previous section, to simulate trap counts obtained by the corresponding baited trap. Since available biological evidence about the dependence of animal movement behaviour on the attractant strength is deficient, we endeavour to consider the broadest range of possible responses by using all possible combinations between different functions *r*(*d*) and *s*(*d*) considered in section “Parameterisation of distance dependence”.

Simulations are performed in a square $L\times L$ domain with $L=50$, with a circular trap of radius $R=5$ placed at its centre. For the sake of simplicity, we assume that animal move in a closed environment, so that they cannot escape from the domain. Correspondingly, a reflecting boundary condition was imposed, so that, if the position of an animal at the next time step (calculated as prescribed by Eq. (1)) appears to be beyond the domain boundary, it is instead placed at the symmetric position inside the domain. The initial population of $N_0=10^4$ individuals was considered and each simulation was run for $1000$ time steps. In order to reveal the generic pattern and to decrease the effects of stochasticity (cf. Fig. 3), each simulation was repeated 30 times and the corresponding average of trap counts at each time step was calculated.

**Fig. 7:**
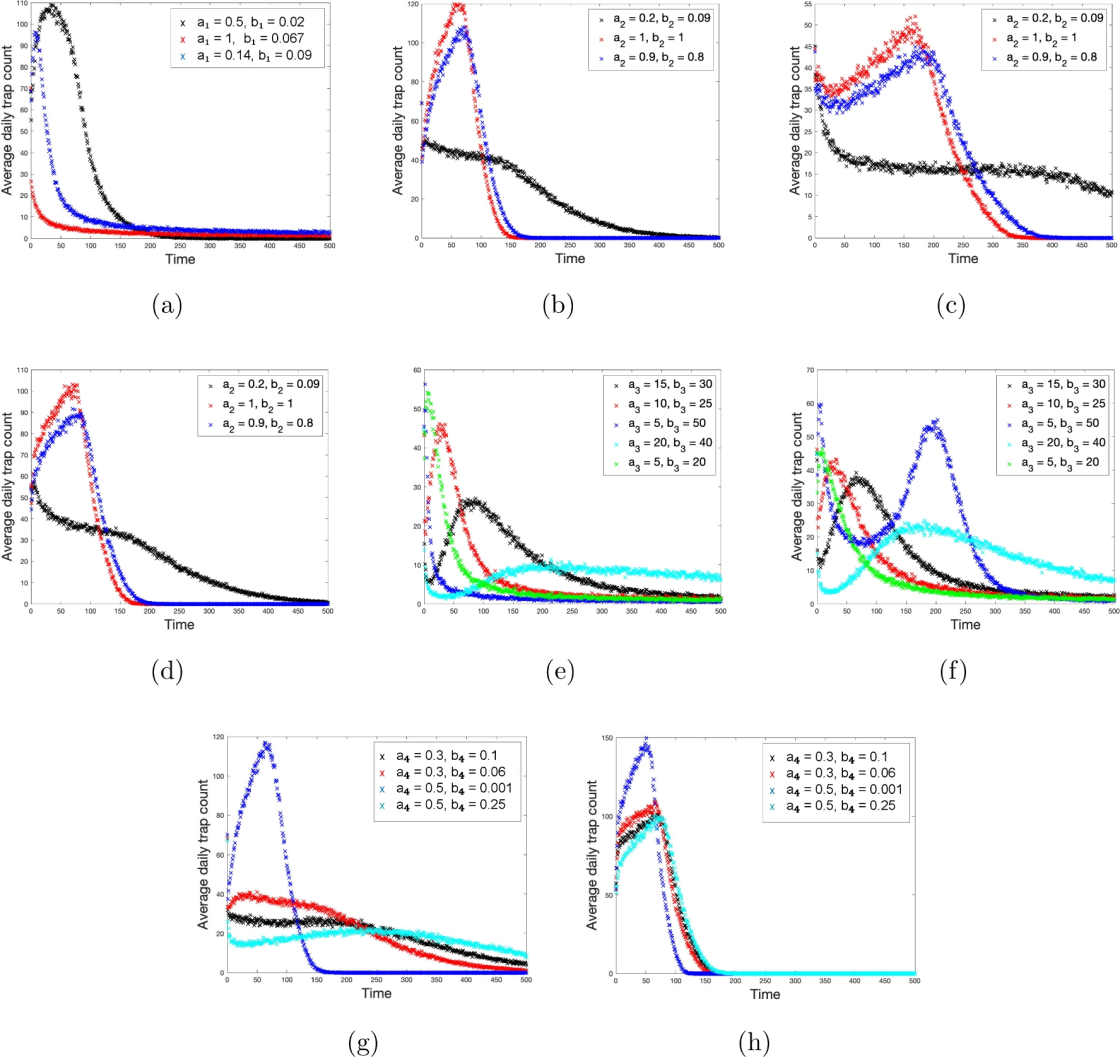
Trap count as a function of time obtained for different combinations of response functions $r_i$ and $s_i$ and for different parameter values (as shown by the figure legends). (**a**) $r_1$ and $s_1$, (**b**) $r_2$ and $s_1$, (**c**) $r_2$ and $s_2$, (**d**) $r_2$ and $s_3$, (**e**) $r_3$ and $s_2$, (**f**) $r_3$ and $s_2$, (**g**) $r_4$ and $s_1$, (**h**) $r_4$ and $s_3$.

Typical trap counts patterns (i.e. trap counts as functions of the discrete time $t_n$) obtained in simulations are presented in Fig. 7. Note that the figure only shows some representative (selected) results; a full collection of obtained results can be found in Supplementary Material. Clearly, the trap count sequences show significant variability in their properties, as is readily seen from the great variety of shapes of the corresponding plots. A closer look, however, reveals that they can be sorted into four qualitatively different distinct patterns:

- Pattern 1. Trap counts decrease monotonically over time; the corresponding graph does not have a kink or inflection point (e.g. see the red curve in Fig. 7a and the blue curve in Fig. 7e).
- Pattern 2. Trap counts show little or no decay for a long period of time, before transitioning to a rapid decay (e.g. see the black curve in Fig. 7c,g). In this case, the corresponding graph has a kink or an inflection point.
- Pattern 3. Trap counts rise from a low initial value to a peak at an intermediate time point and then decay monotonically (e.g. see the black curve in Fig. 7a, the red and blue curves in Fig. 7b,d and all four curves in Fig. 7h).
- Pattern 4. Trap counts initially decrease, then increase to a peak, and then decay monotonically (e.g. see the red and blue curves in Fig. 7c and the blue and cyan curves in Fig. 7f).

More examples for each of the above patterns (obtained for different combinations of the response functions and/or for different parameter values) can be found in the full collection of results available in the Supplementary Material.

We therefore readily observe that the situation where trap counts show an increase over a certain period of time, either at the beginning or at some intermediate time (as in patterns 3 and 4, respectively), is fairly typical. Remarkably, this happens under strictly controlled conditions where the population size (the number of animals in the area around the trap) cannot increase; in fact, the population size is gradually decreasing with time as individual animals are eventually trapped and hence removed from the domain. Thus, the intuitive expectation that an increase in the trap counts indicates an increase in the population density in the vicinity of the trap is entirely wrong. In the case of a baited trap, such increase can happen just as a result of a change in animal movement behaviour due to the effect of the attractant.

For a particular combination of functions *r*(*d*) and *s*(*d*) and specific parameter values, there can also occur some more exotic patterns. For example, the blue curve in the first three panels in the middle row of Fig. A.3 and the blue curve in the third panel in the middle row of Fig. A.4 in the Supplementary Information show two peaks: a narrow peak at a very early time (after just several time steps at the beginning) and a broad peak at a much later, intermediate time.

We note that there can be a considerable variation in the precise form of each of the above patterns such as, for instance, the height and timing of the peaks, the rate of decay, etc. However, a more precise characterisation of such variations caused by changes in parameter values is beyond the scope of this paper. Arguably, a more quantitative insight into such variations would only be sensible if more biological information were available about actual animal’s movement behavioural response to different degrees of the attractant strength.

## Discussion

Traps are routinely used in many ecological and agricultural applications, in particular, for pest insect monitoring as a part of Integrated Pest Management^56^ and invasive species management and control^6^. Understanding of trap counts remains a problem though. Consequently, relating trap counts to a specific population density (more generally, to insects ‘density-activity’, see^57^) in the area around the trap poses a considerable challenge. A consistent approach for estimation of the population abundance from passive trap counts has been developed in Refs.^17,18,52^. In parallel, Ref.^15^ developed a similar mathematical framework to estimate population abundance from active trap counts by treating the attractant radius as the effective trap boundary. This approach has since been further developed, validated, and successfully applied to some field data in Refs.^16,24,40,41^. However, interpretation of trap counts remains more often relative rather than absolute (but see^41^), so that, in the nutshell, a larger trap count is often thought to be an indication of a higher abundance of the monitored population. In applications to a specific pest or specific vegetation (e.g. crops), such interpretation is often justified by relating it to historical data. Correspondingly, some population thresholds are introduced^58,59^: once trap count exceeds a certain number (threshold), it is regarded as an indication that the population density has reached a potentially dangerous level and hence control measures should be applied, e.g. by spreading chemical insecticides.

In this paper, we have challenged the baseline assumption behind trap count interpretation that a larger count would necessarily indicate a larger population. Using a simulation model that allows trap count time sequences to be simulated, we have shown that an increase in trap counts over time may be purely a result of a change in the insect movement behaviour as a response to the attractant. Such a change was indeed observed in many empirical studies, e.g. see^33–35,37–39^, however the understanding of its effect on trap counts, in particular on trap count tendencies over time (i.e. to increase or decrease) remained lacking.

We mention here that, while the presence of an attractant, such as pheromones, light and/or colour, is known to affect insect movement behaviour (e.g. by modifying the average speed of its flight and/or the frequency of turning), the exact effect depends on many factors and hence varies considerably. The movement pattern of some flying insects was shown to depend significantly on the attractant strength (and hence on the distance from the trap) as well as on pheromones composition, in particular showing changes in the frequency of turning or “zigzagging” and even more so for the speed of the flight in the direction of the bait^33,35,38^. For example, in their study on flight tracks of fruit moth (*Grapholita molesta*), Willis and Baker^35^ (see also^60^) found that, as the male moths approached the source, their mean ground speeds decreased and their average rate of turning increased.

Interestingly, in the case of light-baited traps, an increase in light intensity was found to have an opposite effect resulting in an increase in the speed of the flight on the approach to the light source^37^. In fact, not only may different species show a qualitatively different response to an attractant of a different origin (e.g., pheromones, light or colour) but, even to the same attractant. Furthermore, the response of animals of different species but of the same genus can vary significantly, in particular in the effect of the attractant on their movement speed^36^.

Given the complexity and variability of insect movement behaviour in the presence of a bait (as mentioned above), in our study we have attempted to make a broad picture of possible insect responses to the attractant by considering different dependencies of their movement speed and turning angle on the distance from the trap (hence, on the attractant strength); see Eqs. (7)–(10) and (11)–(14). By considering all possible combinations of these archetypal dependencies and using different parameter values, we have simulated a few hundred situations to produce, respectively, a few hundred trap counts sequences; see Figs. A.1–A.4 in Supplementary Information. (For a representative subset of the whole collection, see Fig. 7 above.) This large number of different situations can be sorted into a few qualitatively different patterns of trap count dependency on time; see Patterns 1–4 in “Results” section. We mention here that the diverse array of trap count patterns obtained for baited traps are in stark contrast to the single pattern—a monotonous decay with time—obtained for a ‘passive’ unbaited trap and population of a non-increasing size^17^. This difference is understandable, as the presence of an unbaited trap does not lead to any change in insect movement behaviour.

An interesting question arises here as to how our findings relate to the ‘large unbaited trap concept’, e.g.^15,16^. Indeed, it is well known that, under the assumption of a uniformly distributed initial population, a sequence of trap counts obtained by a single unbaited trap shows a clear trend to decrease with time^17,18^. By contrast, in the present study we have shown that a non-trivial, counter-intuitive behaviour of the simulated trap counts, e.g. their increase with time leading to a peak at some intermediate time, is fairly typical; see Fig. 7 and Figs. A.1–A.4 in Supplementary Information. To resolve this apparent discrepancy, we recall here that the presence of trap introduces a perturbation into the distribution of the population density, and the key process contributing to that is animal movement. However, the re-distribution of the population does not occur instantly; in fact, it can take a considerable time when the spatial distribution is transient, before a long-term ‘asymptotic’ spatial distribution emerges. Formation of the peaks in the trap counts sequences is therefore an essentially transient phenomenon that can be linked to the spatial redistribution of the population, cf. Figs. 2 and 3. Once the initial uniform population distribution has evolved to a heterogeneous one (where the characteristic heterogeneity distance is consistent with the effective attraction radius of the trap), the transient has passed too. Correspondingly, at a later time the baited trap becomes equivalent to a large unbaited trap, so that the trap counts only exhibit a monotonous decay with time, cf.^15,17^. Importantly, the transient spatial population distribution is occurring in the vicinity of the baited trap where animal movement is affected by the attractant, i.e. within the radius of the corresponding large unbaited trap. Thus, the large unbaited trap concept, by its very definition, cannot resolve the transient dynamics and hence is not capable of detecting the transient peaks in the population counts sequences.

Interestingly, at least some of the patterns seen in our simulated trap counts have indeed been observed in field studies, which arguably validates our approach. Trap counts by a baited trap often show a tendency to increase with time to create a peak^61–63^ with a subsequent decay to lower values. This corresponds to our Pattern 3 (see “Results” section). There is one important difference in the underlying factors though. In Ref.^62^, the peak was regarded as an indication of an increase in the population size of the monitored population (of swede midge, *Contarinia nasturtii*). In our study, however, an increase in trap counts occurs solely as a result of a change in insect movement behaviour in response to the attractant, without any increase in the overall population size in the area. A gradual decrease over time in counts obtained by a pheromone-baited trap has also been observed^64^; this broadly corresponds to our Patterns 1 and 2.

Note that, in this paper, we are specifically interested in the effect of changes in the individual animal movement (resulting from their response to the attractant) on the dynamics of trap count. Correspondingly, our focus is on movement and many other processes are deliberately left out of the consideration. For example, in our model the population size cannot increase as the model does not include any processes that can result in that, such as births or immigration. A question therefore remains as to how trap count pattern(s) would change if the population size actually increases with time. Intuitively, one might expect that periods of trap counts increase would become more common and/or the rate of the trap count increase over time could become higher. However, because of the high generic complexity of the dispersal-growth system^65^, intuitive insight is hardly enough and in fact can be misleading. Instead, the issue should be properly investigated by running simulations using an appropriately updated mathematical model. This lies beyond the scope of the present study but should become a focus of future research.

Thinking more specifically about the possible effect of births, we also recall that for many insect species their reproduction is limited to a certain season, which is often quite short. Outside of the reproduction season, the contribution of births to the population size of the monitored population can be neglected. Moreover, during the reproduction season, female insects lay eggs and it usually takes a considerable time before eggs develop into the new generation of adults. However, it is very unlikely that eggs or larvae can be trapped by the same traps as trap adults. Thus, the structured life cycle which is characteristic for insect species gives a further reason to ignore reproduction, especially if trap counts are collected on a shorter timescale.

With regard to the effect of deaths (e.g. as a result of predation by natural enemies), here we refer to earlier studies where it was shown for an unbaited trap that the population size in the vicinity (‘catchment area’) of the trap works roughly as a scaling factor for the individual trap counts^17,18^. Correspondingly, as neglecting deaths results in a higher population size, our simulated trap counts provide an upper bound to those where the deaths would have been accounted for.

We mention here that the tendency in a trap count sequence to increase or decrease over certain periods of time, apart from the population abundance and animal behaviour may also depend on other factors, such as, for instance, the spatial distribution of the monitored population. Even in the case of a much simpler unbaited trap, a change in the trap counts from the characteristic monotonous decrease to a peak can be observed if the trap is installed in a vicinity of a patch of high population density, e.g. see^18^. Recall that, in our simulations, the initial population distribution was assumed to be spatially uniform. Arguably, a heterogeneity of the initial distribution could modify the simulated trap counts considerably, hence adding to the challenge of their interpretation.

For a ‘real’ (not simulated) trap count sequence obtained in the field, it can also be significantly affected by environmental factors, in particular by the weather^66^. For example, any change in the weather conditions that decreases insects movement activity would likely result in a lower trap count, cf.^57^. Seasonality is another important factor^67^. For a pheromone-baited trap, the trap counts can also depend on the direction of the wind^64^. All these add to the complexity of trap counts obtained by a baited trap and should be taken into account in trap counts interpretation.

An essential conclusion of our study is that, unless detailed information about insects’ movement responses to different attractant levels is available, trap counts obtained by a baited trap should be regarded with great care. Unless the purpose of trapping is limited to the question whether a given species is present or absent in a given area, for which baited traps are known to be very efficient, interpretation of trap counts is challenging and can be grossly misleading, giving a false signal that the pest abundance is growing while in fact it does not. Arguably, interpretation of trap counts can be significantly improved if, prior to trapping, some biological knowledge for a given species is available about a typical response of individual movement behaviour to different attractant types (which, in case of pheromone-baited traps, also implies different pheromones composition^33,38^) and, most importantly, to different attractant strengths. Clearly, obtaining such necessary biological data will require significant extra work and considerable resources. Hopefully, it will become a focus of future research.

## Supplementary Information

Supplementary Information.

## Data Availability

All data generated or analysed during this study are included in this published article.
