# Supplementary material for: Using mathematical modelling to highlight challenges in understanding trap counts obtained by a baited trap
Source: Sci Rep. 2025 Mar 13;15:8765. doi: 10.1038/s41598-025-91581-0 (PMC11906655; doi:10.1038/s41598-025-91581-0)
Supplement: Supplementary file 1 — Supplementary Information. [file 41598_2025_91581_MOESM1_ESM.pdf]

Towards Better Understanding of Trap Counts Obtained with a Baited Trap: Insights from a Simulation-based Approach

Omar Mazen Alqubori, Daniel Bearup, Sergei Petrovskii

Supplementary Material

A Full collection of simulations results

A.1 Details of the turning angle parameterisation

Response function	a_i	b_i	q	J_i	Response function	a_i	b_i	q	J_i
Eq. (7)	0.5	$\frac{a_1}{R^2}$	-	-	Eq. (8)	0.9	0.8	-	-
Eq. (7)	1	$\frac{a_1}{3R}$	-	-	Eq. (8)	1	1	-	-
Eq. (7)	0.14	0.09	-	-	Eq. (8)	0.2	0.09	-	-
Eq. (9)	15	30	-	0.01	Eq. (10)	0.8	0.1	1	0.01
Eq. (9)	10	25	-	0.01	Eq. (10)	0.3	$\frac{a_4}{R}$	1	0.01
Eq. (9)	5	50	-	0.01	Eq. (10)	0.5	0.001	1	0.01
Eq. (9)	20	40	-	0.01	Eq. (10)	0.5	a_4^2	1	0.01
Eq. (9)	5	20	-	0.01	-	-	-	-	-
Eq. (9)	15	30	-	0.01	Eq. (10)	0.3	0.1	1	0.01
Eq. (9)	15	30	-	0.005	Eq. (10)	0.3	0.1	2	0.01
Eq. (9)	15	30	-	0.002	Eq. (10)	0.3	0.1	3	0.01

Table A.1: Parameter values resulting in a representative variation in the movement behaviour (as is quantified by variation in the randomness parameter) over the space, i.e. the distance from the trap). The corresponding functions $r_k(d)$ $(k = 1, 2, 3, 4)$ are shown in Figs. 4 and 5 in the main text.

A.2 Details of the step size parameterisation

Response function	c_i	h_i	d_{max}	Response function	c_i	h_i	d_{max}
Eq. (11)	6	0.01	-	Eq. (12)	20	0.3	-
Eq. (13)	100	0.3	-	Eq. (14)	10	5	22.5

Table A.2: Parameter values resulting in a representative variation in the movement behaviour (as is quantified by variation in the step length parameter) over the space, i.e. the distance from the trap). The corresponding functions $s_m(d)$ $(m = 1, 2, 3, 4)$ are shown in Fig. 6 in the main text.

A.3 Simulation results for different combinations of the turning angle and step size parameterisation

The figures below provide a comprehensive account of the simulation results obtained in this study by combining different distance-dependence for the turning angle and step-size; see Eqs. (7-10) and (11-14) in the main text.

As is discussed in the main text, a visual investigation of the results readily reveals that the observed trap count patterns can be sorted into the following four distinctly different patterns:

Pattern 1. Trap counts decrease monotonically over time; the corresponding graph does not have a kink or inflection point (e.g. see the red curve in the first three panels in the top row of Fig. A.1 and red, black and cyan curves in the first three panels in the bottom row of Fig. A.4).

Pattern 2. Trap counts show little or no decay for a long period of time, before transitioning to a rapid decay (e.g. see black curves in the first three panels of the second row of Fig. A.1 and red and blue curves in the first three panels of the bottom row of Fig. A.2). In this case, the corresponding graph has a kink or an inflection point.

Pattern 3. Trap counts rise from a low initial value to a peak at an intermediate time point and then decay monotonically (e.g. see red and blue curves in the first two columns of the second row of Fig. A.1 and blue curves in the first two columns in Fig. A.4).

Pattern 4. Trap counts initially decrease, then increase to a peak, and then decay monotonically (e.g. see cyan curves in the third row of Fig. A.1 and blue curves in the three left panels in the middle row of Fig. A.3).

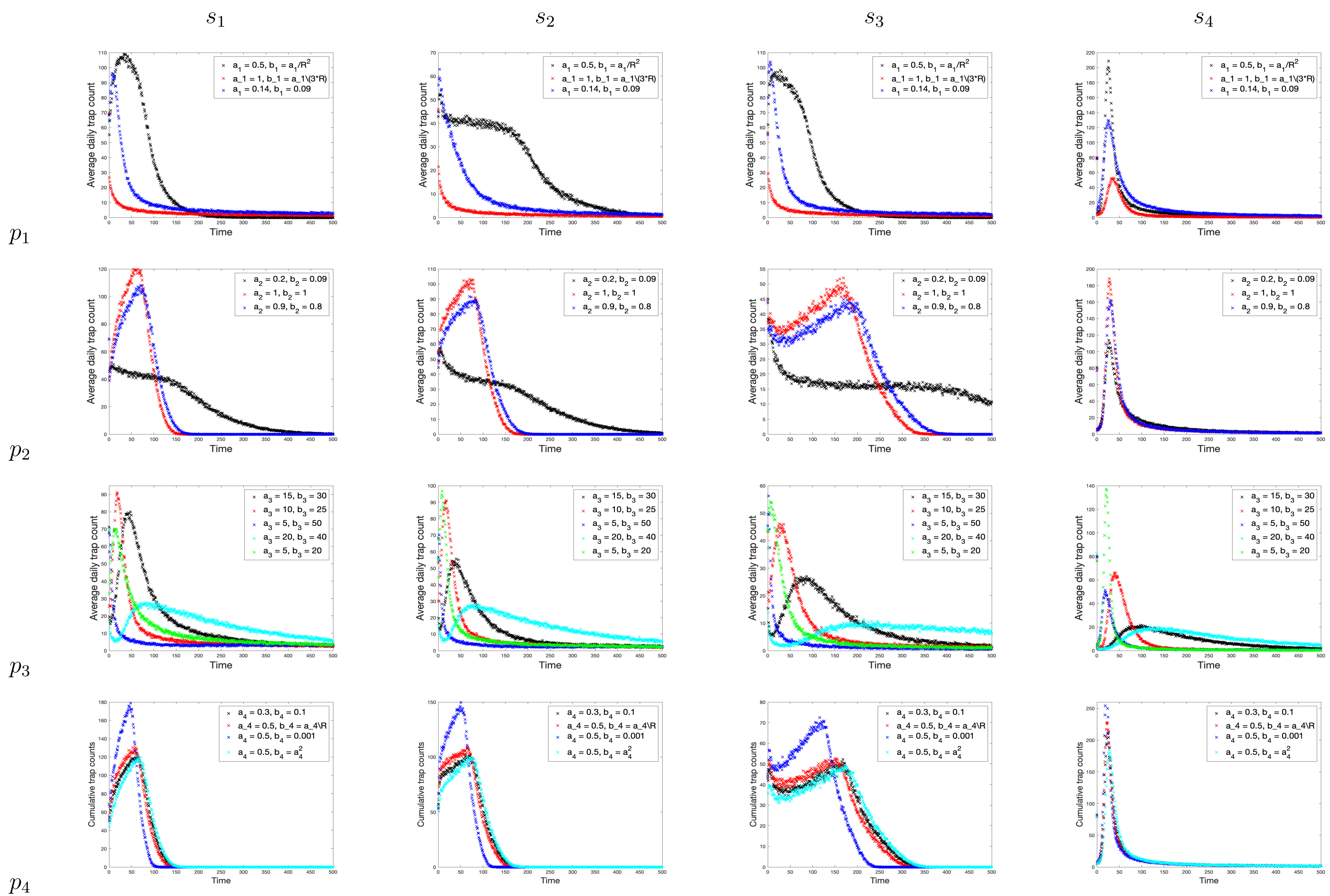

Figure A.1: Average trap counts (from 30 realizations) for different combinations of response functions $r_k(d)$ and $s_m(d)$ (k,m=1,2,3,4). Parameters are given in Tables A.1 and A.2 and in the insets.

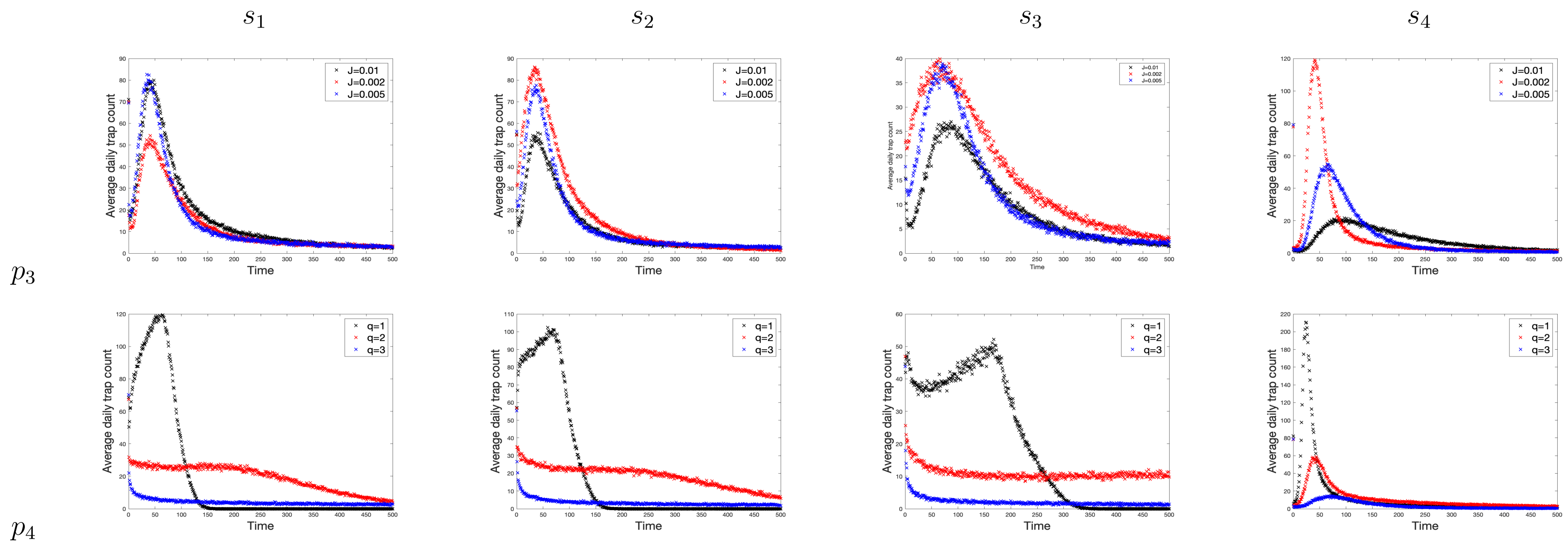

Figure A.2: Similar to Fig. A.1 but with a focus on the effects of changes in parameters J_3, J_4 and q (see Eqs (9) and (10) in the main text) on the average trap counts. Other response function parameters are given in the last 3 rows of Table A.1 and in Table A.2.

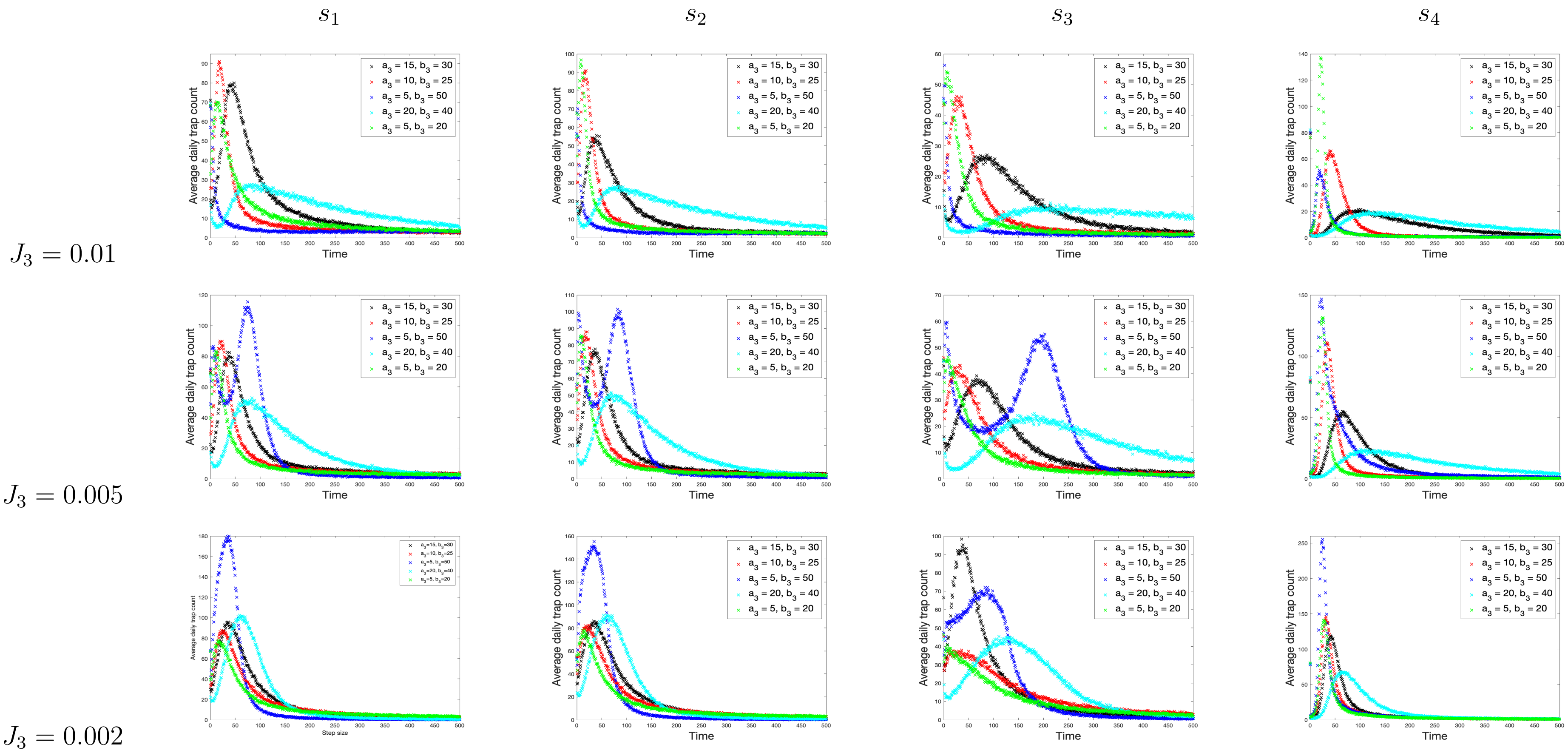

Figure A.3: Similar to Figs. A.1 and A.2, with a focus on the effects of further changes in parameter J_3 (cf. Eq. (9) in the main text) on average trap counts. Other response function parameters are given in the inset captions, in the first block of Eq. (9) in Table A.1 and in Table A.2.

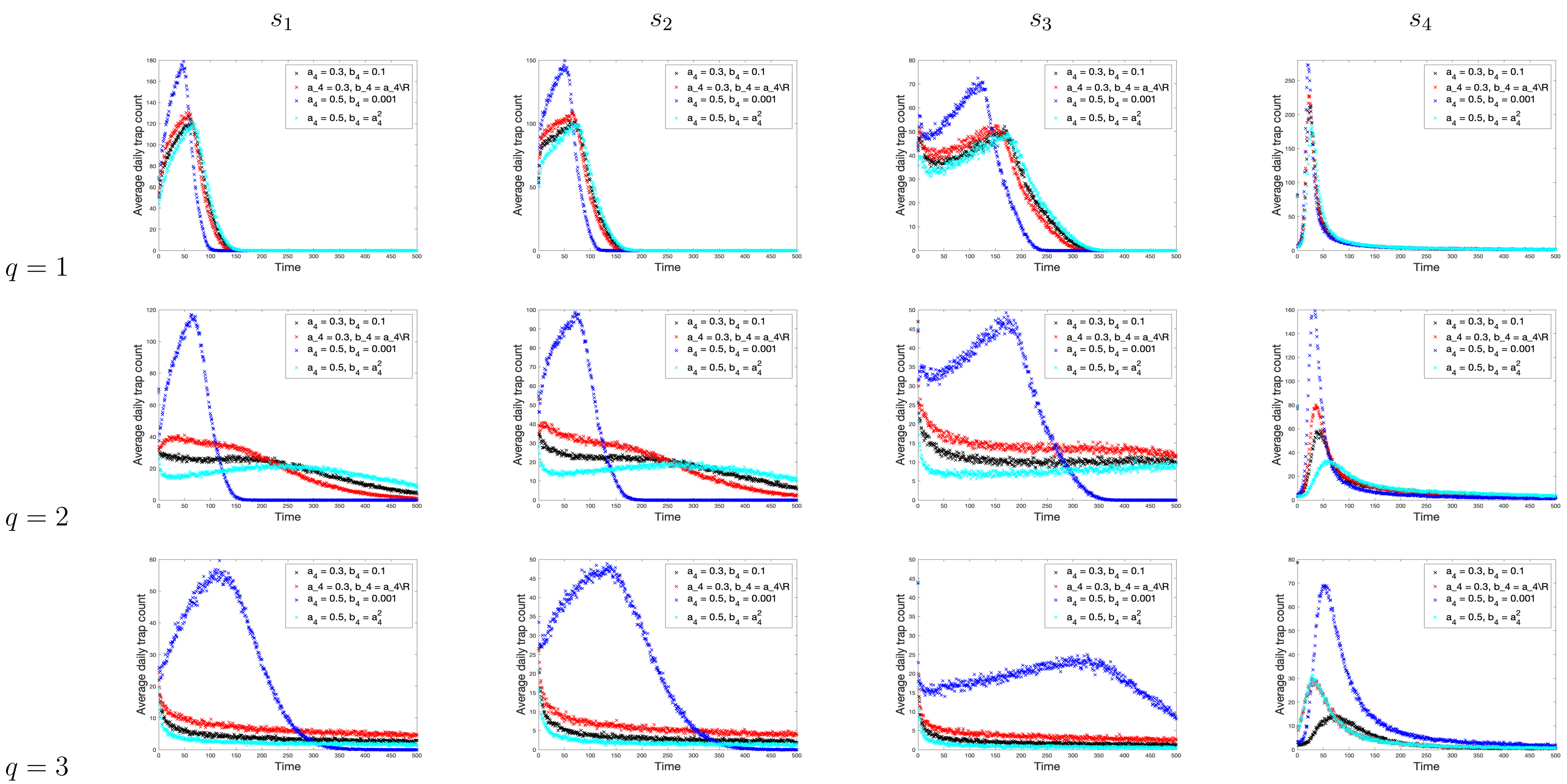

Figure A.4: Similar to Fig. A.1 but now with a focus on the effects of changes in parameter q, cf. Eq. (10) in the main text, on average trap counts. Other response function parameters are given in the first block of Eq. (10) in Table A.1, in Table A.2, and in the insets.
